# Inflammation markers in cutaneous melanoma - edgy biomarkers for prognosis

**DOI:** 10.15190/d.2015.30

**Published:** 2015-03-27

**Authors:** Monica Neagu, Carolina Constantin, Georgiana Roxana Dumitrascu, Andreea Roxana Lupu, Constantin Caruntu, Daniel Boda, Sabina Zurac

**Affiliations:** ^1^Immunobiology Laboratory, "Victor Babes" National Institute of Pathology and Biomedical Sciences, Bucharest, Romania; ^2^Faculty of Biochemistry, University of Bucharest, Romania; ^3^Dermatology Research Laboratory, "Carol Davila" University of Medicine & Pharmacy, Bucharest, Romania; ^4^Department of Pathology, "Carol Davila” University of Medicine and Pharmacy, Bucharest, Romania; ^5^Colentina University Hospital, Bucharest, Romania

**Keywords:** inflammation, melanoma, tissue biomarkers, circulatory immune cells

## Abstract

There is a fine balance between inflammation and tumorigenesis. While environmentally induced inflammatory condition can precede a malignant transformation, in other cases an oncogenic change of unknown origin can induce an inflammatory microenvironment that promotes the development of tumors. Regardless of its origin, maintaining the inflammation milieu has many tumor-promoting effects. As a result, inflammation can aid the proliferation and survival of malignant cells, can promote angiogenesis and metastasis, can down-regulate innate/adaptive immune responses, and can alter responses to hormones and chemotherapeutic agents. There is an abundance of studies unveiling molecular pathways of cancer-related inflammation; this wealth of information brings new insights into biomarkers domain in the diagnosis and treatment improvement pursue. In cutaneous tissue there is an established link between tissue damage, inflammation, and cancer development. Inflammation is a self-limiting process in normal healthy physiological conditions, while tumorigenesis is a complex mechanism of constitutive pathway activation. Once more, in cutaneous melanoma, there is an unmet need for inflammatory biomarkers that could improve prognostication. Targeting inflammation and coping with the phenotypic plasticity of melanoma cells represent rational strategies to specifically interfere with metastatic progression. We have shown that there is a prototype of intratumor inflammatory infiltrate depicting a good prognosis, infiltrate that is composed of numerous T cells CD3+, Langerhans cells, few/absent B cells CD20+ and few/absent plasma cells. Circulating immune cells characterized by phenotype particularities are delicately linked to the stage melanoma is diagnosed in. Hence circulatory immune sub-populations, with activated or suppressor phenotype would give the physician a more detailed immune status of the patient. A panel of tissue/circulatory immune markers can complete the immune status, can add value to the overall prognostic of the patient and, as a result direct/redirect the therapy choice. The future lies within establishing low-cost, affordable/available, easily reproducible assays that will complete the pre-clinical parameters of the patient.

## SUMMARY

1. Introduction
2. Linking inflammation to tumorigenesis**
3. Melanoma inflammatory infiltrate - wolf in a sheep skin?
  - 3.1 Cells that sustain the inflammatory milieu
  - 3.2 Inflammatory molecules expression in melanoma tissues**
4. Inflammatory markers in blood circulation - what do they reflect?
  - 4.1 Circulating immune cells
  - 4.2 Circulatory inflammatory markers
5. Conclusive remarks

## 1. Introduction

Inflammation is a complex cascade of immune, non-immune cells and mediators having as final goal damaged tissue restoration. Inflammation, a subject intensively published at the beginning of the last century^1^, is a process that can be described as three stages or eight stages progression, but whatever the number of described stages is, the process will start with the injury inflicted upon a tissue and it will end with the reconstruction of the damaged tissue.

Inflammation process starts with an injury that impairs tissue structure and that can be generated by a macro- or a micro- physical trauma (e.g. overuse, friction, sun burn). Immediately following injury, ultrastructural changes appear due to the disruption of the cell’s membrane, releasing therefore the intracellular contents into extracellular space. Hypoxic-related metabolic changes occur, cells become deprived of oxygen (secondary hypoxic injury), sodium pump fails, hence intracellular sodium increases, cell membrane disruption continues in adjacent cells and intracellular contents is once more spilled out. An extracellular cascade is generated; mediators (e.g. histamine, bradykinin) are the first signals that trigger an inflammatory response. While the inflammatory response is initiated, hemodynamic changes occur: arteries dilate enhancing blood flow, inactive capillaries and venules open, total blood flow increases, rate of flow decreases and leukocytes, otherwise in the blood stream, start to adhere to the vessel wall. Permeability changes, gaps develop in the vessel walls and leukocytes transmigrate to the injured site. Leukocytes migrate guided by the chemoattractants gradient concentration. First to arrive at the injured site are neutrophils, these cells being the *temporary* first line of defense because they are short lived cells (approx. 7 hours); they are followed by macrophages that build up the second line of defense and can live herein for months. These two phagocyte type cells process cellular debris/microbes/triggering agents within the inflamed tissue and enhance the clearance process through lymph vessels^1,2^.

**Figure 1:**
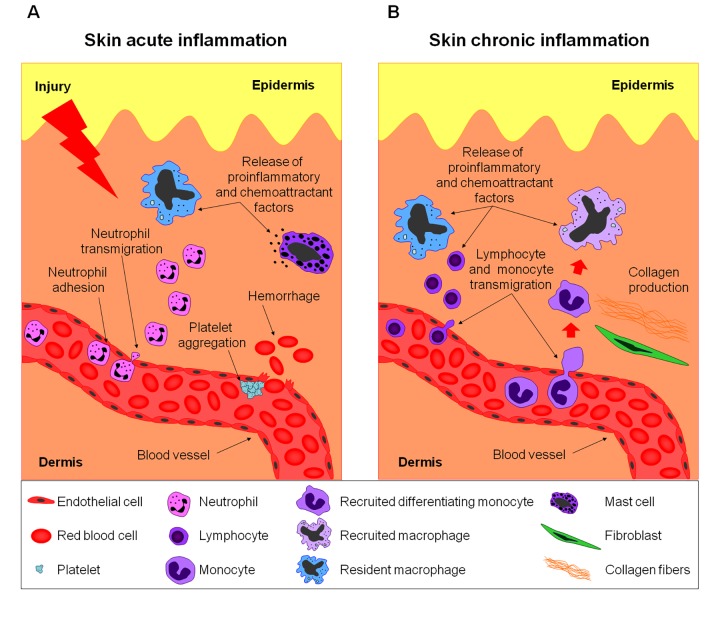
Acute and chronic skin inflammation cascade ****A.**** The initial skin injury triggers first intravascular processes that activate neutrophils’ adhesion and transmigration. Resident macrophages and mastocytes release pro-inflammatory and chemoattractant factors; **B.** Lymphocytes and monocytes have increased adhesion capacities and further transmigrate into extravascular space. Transmigrated cells and resident macrophages secrete pro-inflammatory and chemoattractants factors, stimulating collagen production and perpetuating the inflammatory response.The HIV-1 PR is shown as a cartoon in green. The key residue mutations contributing to flap dynamics are shown in red. The catalytic aspartic acid residues are shown in blue. Darunavir is bound to the active site in cyan.

There are key issues in inflammation, meaning that acute and chronic stages of this process interwind (**Figure 1**). In this view, and in the framework of our paper, the scientific trend is to incriminate chronic inflammation as linked to carcinogenesis.

During acute inflammatory responses, innate cells secrete mediators that attract Th1-polarized T lymphocytes, these lymphocytes secrete cytokines with antitumor potency (e.g. IL-2 and IFN-gamma). T cells in combination with antitumor-directed B-cell-derived factors (e.g. immunoglobulins - Igs) activate tumor inhibitory responses sustained by newly recruited innate immune cells and effectors cytotoxic T lymphocytes (CTLs); all these *cellular* *soldiers* comprise an *army* that can induce a tumor rejection.

In contrast, when there is a chronic activation of immune response without any resolution of the damaged tissue, accumulation of regulatory T (Treg) cells, Th2 cells, and activated B cells is induced; these cells secrete pro-tumorigenesis factors (e.g. IL-4, IL-6, IL-10, IL-13, transforming growth factor beta - TGF-beta) that enhance pro-tumorigenesis responses in innate immune cells and inactivate CTL cytotoxicity, processes that favor tumor promotion^3^.

Consequently, mediators and cellular effectors of inflammation are common to tumor microenvironment as well.

Inflammatory conditions can preclude a malignant transformation and/or an oncogene alteration can sustain the inflammatory microenvironment favorable for tumor development^4^.

We will tackle herein the inflammatory markers, whether tissue related or soluble ones that can pinpoint the prognosis of a deadly skin cancer, like melanoma.

## 2. Linking inflammation to tumorigenesis

In cutaneous tissue there is an established link between tissue damage, inflammation, and cancer development. Inflammation is a self-limiting process in physiological conditions while tumorigenesis is a complex mechanism of constitutive pathway activation^5^.

As stated in the introduction, skin’s chronic inflammation can be one of the traits for tumor initiation and progression. Long-term production and accumulation of inflammatory factors like cytokines/chemokines can induce both locally and systemically an immunosuppressant milieu further associated with cancer progression.

However, in melanoma, the correlation between inflammatory mediators, immunosuppressive cells and clinical outcome of the patient is still a matter of intense research. Recent studies are foreseeing myeloid-derived suppressor cells (MDSCs) and Tregs as important prognostic biomarkers for high risk disease progression^6^.

Cytokines, long-time players in inflammation, were recently linked to melanoma tumorigenesis. In the skin, cytokines are produced by resident cells (keratinocytes, Langerhans cells - LC, melanocytes, mast cells - MC, macrophages), but as well by, inflammatory recruited cells: neutrophils, eosinophils and lymphocytes^7^.

Cytokines are, in general, not stored in the cells, but are synthesized following cell activation. These molecules are a very heterogeneous class of molecules; they include lymphokines, monokines, interleukins, interferons, growth factors and chemokines. Cytokines, act locally within the tissue, having a paracrine function on neighboring cells that express specific receptors or have an autocrine action on the producing cells - auto-regulatory loop.

When there is a prolonged inflammatory stimulus, cytokine production is excessive, and besides having a deleterious effect on the inflamed tissue they can affect cells distant from the place of the initial inflammation, an action resembling with that of hormones. Moreover, the cytokine receptors are often homologous, hence an array of various cytokines have multidirectional pleiotropic effects. Beyond that, cytokines can have a synergistic effect on one cell type, and/or act antagonistically on other cell type.

**Figure 2:**
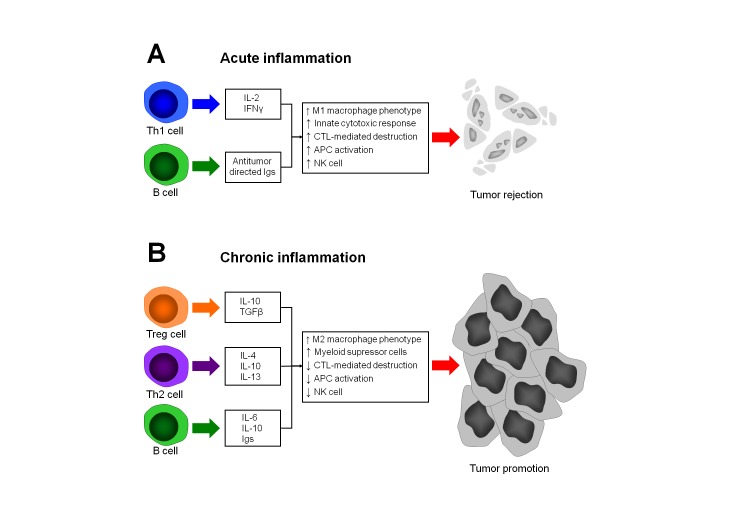
Acute and chronic inflammation elements that are linked to tumorigenesis ****A.**** T and B lymphocytes secrete factors that induce M1 macrophage phenotype, enhance innate immunity, CTL-mediated destruction, activation of the antigen presenting capacity and NK cell activation. All these processes have a potent anti-tumorigenesis action; **B.** T and B lymphocytes secrete factors that induce M2 macrophage phenotype, enhance myeloid suppressor activity, reduce CTL activity, decrease antigen presenting capacity and NK cell activity. All these processes have a potent pro-tumorigenesis action.

Being acknowledged that, in each tissue, a complex and precisely regulated cytokine network is developed^8^, a cascade that can trigger a tumorigenesis process is mounted (**Figure 2**).

Cell’s migration is important and critical for several processes such as normal embryogenesis, immune response, inflammation, but as such, is one of the key events in cancer metastasis^9^. Repetitive UV exposure of primary cutaneous melanomas in genetically engineered mouse model induces metastatic progression, independent of its tumor-initiating effects. The metastatic event was dependent on the neutrophils’ migration, recruitment and activation. This clear inflammatory process was initiated by the release of high mobility group box 1 (HMGB1) from UV-damaged epidermal keratinocytes and conveyed by Toll-like receptor 4 (TLR4). The inflammatory response to UV mediated by neutrophils further stimulated angiogenesis and additionally activated melanoma cells to migrate towards endothelial cells.

These recently published results have shown that UV irradiation directly acts on epidermal keratinocytes, cells that in turn activate innate immune system. The resulting inflammatory response increases non-immune responses, hence melanoma-endothelial cell interactions leading to perivascular invasion (in histopathology the term is used as “angiotropism of human melanomas”).

These results emphasize once more that ulcerated primary human melanomas abounding in neutrophils and displaying reactive angiogenesis have a high risk for metastases^10^.

Molecular perturbations underlying non-healing wounds and chronic inflammation are yet again the centre of scientific research. The effect of a novel cancer promoter (Ehm2), in wound healing and its link to inflammation was reported. Ehm2 belongs to the FERM family of proteins (Four.1 protein, ezrin, radixin, moesin), family that is involved in membrane-cytoskeletal interactions, and linked to the metastasis event in several cancer types, including melanoma.

In a recent study, the effect of Ehm2 knockdown on migration, adhesion, growth, cell cycle progression and apoptosis was reported. The authors show that Ehm2 expression is three times higher in acute inflamed tissues, compared to the chronic state. Increased Ehm2 expression matches healing, especially at the leading wound edge. When Ehm2 was knocked-down, several cellular processes were hindered; as such reduced cellular adhesion, migration/motility, without affecting growth, cell cycle and apoptosis were reported. Also Ehm2 knockdown induced another reduced protein, neural Wiskott-Aldrich syndrome protein (Nwasp) expression^11^. This Nwasp is important because the binding of cortactin to Arp2/3 and Nwasp are key elements for invadopodium formation in melanoma cells^12^. The reported results suggest that Ehm2 can be an interesting bridge between inflammation and melanoma metastasis, its knockdown down-regulating the expression of Nwasp, through which it may exert its effect on cellular migration^11^.

We can draw some common molecular pathways for both wound healing and tumorigenesis. Thus, both processes use signaling pathways, like Ras, Hedgehog and WNT and were reported as deregulated in wound healing and tumorigenesis^13^.

Different cell types interact intimately; epithelial cells, mesenchymal stem cells and immune cells interact in order to develop the complex process of inflammation subdued in wound repair or in tumor formation. All the developed changes upon tissue injury in the cellular microenvironment can induce the development of a tumor. Clinicians report how a 60 years' chronic foot ulcer could lead to a foot melanoma in a 77 years old patient^14^.

Epithelial-mesenchymal transition (EMT) is a recent reported process through which epithelial cells lose some of their innate characteristics (cell polarity and cell-cell adhesion) and are endowed with migratory and invasive properties becoming mesenchymal stem cells^15^. Wound healing has remarkable similarities to cancer in EMT induction^16^. During wound re-epithelialization epidermal keratinocytes, acquire migratory phenotypes^17^. In wound healing, the migratory potential of epithelial cells returns to normal upon wound closure, during the rebuilding of basement membrane. In tumors, these processes are uncontrolled, epithelial cells can harbor oncogenic muta­tions leading to immortalization, to EMT initiation and cancer stem cells properties acquisition^18^. Trans-differentiation is a recent discovered cellular process in which, a mature somatic cell is transformed into another mature somatic cell without an intermediate pluripotent state or progenitor cell type^19^**. **There is still lit­tle evidence that lineage conversion from one cell type to another occurs to a substantial extent in adult tissues, but regarding mesenchymal stem cells they have been reported to undergo trans-differentiation into epi­dermal cells, endothelial cells and pericytes, particularly following wounding^20,21^.

In experimental B16 melanoma tumors, it was recently demonstrated that pericytes are the major sources of the secreted glycoprotein and integrin ligand lact-adherin (MFG-E8). MFG-E8 promotes angiogenesis *via* enhanced PDGF-PDGFR-beta signaling mediated by integrin-growth factor receptor crosstalk. The actual role of MFG-E8 in skin physiology was recently studied and reported. Thus, in normal murine and human skin dermis, accumulations of MFG-E8 were identified around CD31(+) blood vessels, co-localized with PDGFR-beta(+), alphaSMA(+) and NG2(+) pericytes. Inflammation during wound healing was characterized by high expression of both MFG-E8 protein and its mRNA. In MFG-E8 knockout mice wound healing was delayed, process that was associated with reduced myofibroblasts and vessel numbers in would areas. MFG-E8 promotes cutaneous wound healing by enhancing angio-genesis and, as a result, this enhanced angiogenesis can switch to an eventual pro-tumor action^22^.

Another mechanism that can link inflammation with tumorigenesis is the fact that during wound heal­ing, fibroblasts deposit excess collagen (fibrosis stage), which leads to scar formation. This fibrotic connective tissue is a tumor permissive microenvironment^23^.

In the microenvironment there are remarkable similarities between the bulk of growth factors, cytokines and chemokines present in healing wounds and in tumors, the slight differences reside in the expression kinetics^4^.

If one intends to prevent any tumor development at wound sites, the logical clinical approach is to block inflammation (see also **Figure 2**).

In conclusion we can state that there is a close relationship between chronic tissue damage, inflammation and cancer^5^; tumors can develop, though infrequently, at the site of chronic skin wounds^24^. Moreover targeting inflammation and coping with the phenotypic plasticity of melanoma cells represents rational strategies to specifically interfere with metastatic progression^10^. An efficient wound repair is crucial for skin’s homeostasis, while a defective wound repair inclines toward skin’s tumorigenesis.

## 3. Melanoma inflammatory infiltrate - *wolf in a sheep's skin*?

Although an intense studied subject, the prognostic value of an inflammatory infiltrate in melanoma is still a matter of debate. The major debated subjects incline to the role of inflammatory immune cells within the tumor: are they markers for a local immune anti-tumoral activity or are they “converted” towards pro-tumoral activity?^25^

### 3.1. Cells that sustain the inflammatory milieu

There are several cells that gained research momentum in the last years regarding their biomarker potency in melanoma. One of these cells is the macrophage that is a myeloid cell playing an essential role in inflammation and host defense, regulating immune responses and maintaining tissue homeostasis. Depending on the microenvironment, macrophages can polarize to two distinct phenotypes. The M1 phenotype is activated by IFN-gamma, and displays an inflammatory profile, while M2 macrophages are activated by IL-4 and tend to be anti-inflammatory or immunosuppressive^26^. Melanomas that have lost the specific MelanA expression are hard to diagnose in comparison to tumors of mesenchymal origin. Morphological changes leading to mesenchymal shape and cellular de-differentiation can induce developmental programs (e.g. EMT) and disseminative tumor cells. In this aspect, inflammation process and macrophages CD163(+) are inducers of E-cadherin and cell-to-cell adhesion loss, events that govern EMT. Results published in 2015 obtained from studying a large cohort of melanoma patients, showed the existence of MelanA-negative clones and MelanA-positive clones in tumor tissues. MelanA-negative clones correlated significantly with an augmented inflammatory response of tumor-infiltrating macrophages CD163(+), complete loss of E-cadherin and a spindle-shaped morphology, irrespective of ulcerated status. The inflammatory heterogeneity of melanoma, with important diagnostic, prognostic and therapeutic biomarker potency, resides in cell clones resembling tumor-associated macrophages. This inflammatory phenotype of macrophages is associated with loss of MelanA expression, possible marker of a more invasive tumor cells^27^.

Classic inflammation players, like complement system elements, were also studied in relation to tumorigenesis. A recent interesting paper emphasized the importance of C5a in cancer, especially in melanoma. In the immune system, complement is involved in several mechanisms, but recently one of its components, C5a may also serve to potentiate cancerous process. C5a is known as a potent chemoattractant, hence it facilitates cellular proliferation and regeneration by attracting MDSCs and supporting tumor promotion^28^.

For the first time, in 2012 it was shown that C5a, plays an inhibitory role for conventional dendritic cells (DCsc)-mediated NK-cell activation. The presented findings show that C5a-induced TGF-beta1 production by Gr-1+ myeloid cells and it regulates cDC-NK-cell activation, this being a previously unidentified mechanism of immune regulation. There is a new model of complement-regulated cDC-NK-cell activation in which an immune marker, a complement component controls host’s response to danger^29^. Previous to this study, a delayed tumor growth was obtained in mouse models of melanoma in complement deficient and C5aR antagonist-treated mice. This phenomenon was shown to be a consequence of defective MDSC function and reliant upon activation of CD8(+) T cells^30^.

Analyzing over 100 melanoma patients, we have shown that the inflammatory infiltrate in regressed and non-regressed tumor components, have different distribution of inflammatory cells^31^. Inflammatory infiltrate consists mainly of T lymphocytes CD3(+) as previously reported^32,33^. A significant association between high pT level and presence of frequent CD3(+) T cells and ulceration was identified^33^. Non-ulcerated tumors have similar distributions of CD3(+) cells irrespective of pT level; significantly more numerous cases with ulceration presented frequent CD3(+) cells in association with high pT levels. Our reported findings are surprising, considering that the overall favorable prognostic significance associated with brisk tumor infiltrating leukocytes (TIL)^34-40^. Our data indicate the presence of abundant TILs within thick ulcerated tumors (unfavorable prognosis). In our opinion, more likely this infiltration represents a normal enhancement of the inflammatory infiltrate within an ulcerated tumor as a physiologic reaction to ulceration^31^.

The activation degree of T lymphocytes CD4(+) was investigated evaluating membrane CD134 expression (OX40), this molecule being a member of the TNFR-superfamily expressed on activated T cells. It was reported that CD134 expression has been associated with favorable cancer patient outcomes. The percentage of CD134 marker on CD4(+) T cells from SLNs *versus* peripheral blood lymphocytes was related indirectly to the T stage of the primary tumor and was reported as decreased in ulcerated primary tumors and positive sentinel nodes. Activation decreases in more advanced tumor features (higher T stage, ulceration) and nodal involvement, hence an immunosuppressive effect on the SLN microenvironment^41^.

In the cases we have investigated, B lymphocytes CD5(+) presented similar distribution as T CD3(+) in inflammatory infiltrate. When analyzing mature T cells CD7(+) we have seen an overall tendency of losing CD7(+) expression in inflammatory infiltrate both intratumor and in regression areas. At tumor site there is a slight predominance of CD4(+) cells uncorrelated with pT level, ulceration or regression^31^.

B lymphocytes CD20(+) are increased in pT4 tumors and prevail in ulcerated tumors. Presence of B cells within TIL was previously identified^42^, and correlated with better prognosis^43^. In the cases we have published, presence of more numerous B cells in ulcerated tumors may be secondary to ulceration (as part of subsequent inflammatory reaction) and therefore should not be regarded as indicator of bad prognosis. Plasmocytes CD138(+) were present in almost all areas of regression and, when present, they were frequent, irrespective of regression type or ulceration^31^.

A subset of B-lymphocytes (B-1) was reported as having *in vivo* pro-metastatic effects on melanoma cells through a direct cell-cell interaction. Identifying this B-1 subpopulation in tumor samples, a direct correlation with MUC18 expression in melanoma cells was reported. Besides the fact that this sub-population of lymphocytes can indicate a metastatic process, MUC18 expression can be therapeutically triggered in human melanoma, reducing therefore the invasion event^44^.

Antigen presenting cells, are one of the key players in of the adaptive immune response and, in this matter, DCs are one of the most potent activators of the immune response against tumors.

Langerhans cells (LC) (CD1a+ Langerin+), specific DCs, are major cells in skin’s immune system and subject of intense research in melanoma. LCs are phenotypically mature^45^, but, surprisingly, functionally defective in melanoma negative SLNs. LCs are the most represented DC subset in melanoma’s SLNs. Recently it was reported that LCs from both negative and positive SLNs, have a lower expression of CD83, CD80, CD86, and HLA-DR compared to LCs migrated from epidermal explants and, surprisingly, similar to the expression observed in freshly isolated epidermal LCs. The percentage of LCs expressing CD83 in positive SLNs was significantly lower than the percentage found in negative SLNs whereas that of LCs expressing CD80, CD86, and HLA-DR did not differ significantly.

Tissue immune markers like the specific LC phenotype can indicate a highly immunosuppressive microenvironment^46,47^ and hence a poor clinical outcome. The authors highlight a very interesting immune mechanism in which LCs in melanoma patients migrate from the skin to SLNs having an immature phenotype, this phenotype induces a tolerogenic milieu for melanoma associated antigens^48^.

When we have studied our group of patients, we have scarcely found LC within tumor mass, and, when present, LCs were negatively correlated with Breslow index (higher the pT level, more probable absence of LC), but not with ulceration^31^. However, when correlating LC and pT level separately in ulcerated and non-ulcerated tumors, we have identified their presence in thinner tumors. Presence of LC within the tumor is associated with factors of better prognosis (thinner tumors), most likely these cells being involved in antitumor host defense by presenting antigens to CD8(+) cells^49^. The maturation state of DCs in cutaneous melanoma can be prognosticator biomarker in this disease. The density of DCs expressing CD1a and the maturation marker DC-LAMP was determined in primary tumors. CD1a(+) DCs were found infiltrating both melanoma cell nests and the surrounding stroma, while DC-LAMP(+) mature DCs were generally confined to the peritumoral areas, associated with lymphocytic infiltrates. DC density was associated with activated (CD25(+) / OX40(+)) T lymphocytes while infiltration of CD1a(+) and DC-LAMP(+) DCs were negatively correlated with melanoma’s thickness. High peritumoral density of mature DCs was associated with longer survival, and combination of high peritumoral CD1a(+) or DC-LAMP(+) cell density with high number of CD25(+) or OX40(+) lymphocytes pin-pointed a patients subgroup with more favorable survival. High DC-LAMP(+) cell/high OX40(+) cell density and Breslow index are reported as independent predictors of good prognosis. The density of mature DCs, especially in association with activated T cells, have prognostic importance, and can be indicators of a functional immune response and hence a better outcome of the disease^50^.

In a large patients’ cohort (more than 2,000 patients followed for almost 8 years), recent published results focused on factors known to modify systemic inflammation (low vitamin D levels, high body mass index, use of aspirin or nonsteroidal anti-inflammatory drugs or smoking). All these parameters were tested as predictors for melanoma-specific survival (MSS). This study brought evidence that lower vitamin D levels and smoking at diagnosis are associated with ulceration of primary melanomas and a lower MSS^51^.

Another study extended the prognostic impact of ulceration to the adjacent epidermal involvement, subsequent to the inflammation (re-epithelialization and reactive epidermal hyperplasia). In over 380 patients, the presence of an attenuative type of ulceration and excessive ulceration were found as independent predictors of poor melanoma survival. Studying the epidermal involvement of the surrounding epidermis, authors show that the extent and type of ulceration along with the involvement of the surrounding epidermis increase the prognostic information for melanoma survival^52^.

### 3.2 Inflammatory molecules expression in melanoma tissues

The overall expression of inflammatory molecules can drive the tumor invasiveness. When studying the correlation between the expression of some melanocyte differentiation antigens (e.g. alpha-MSH, Melan-A, gp100) and several immune molecules expression, such as adhesion molecules and cytokines, interesting results were found as correlated with patient’s survival.

Lymph node samples were studied in patients receiving autologous TIL plus IL-2 as immunotherapeutical agents. A low expression level of TGF-beta, IL-10, ICAM-1 and alpha-MSH expressed by tumor cells were significantly associated with a prolonged relapse-free survival and a longer overall survival. Immunosuppressive cytokines (IL-10, TGF-beta) and specific hormones (alpha-MSH) could be markers for favorable prognostic in patients receiving immune therapy^53^.

There is a clear immune suppressive milieu developed at tumor site where several inflammatory cells and molecules convey to tumor development^54^. Macrophages secrete indole amine 2,3-dioxygenase (IDO) that induce an inhibition of T-cell proliferation due to tryptophan depletion and, moreover, IDO recruits Tregs FOXP3(+) into the developing tumor. Recruiting more TGF-beta-secreting Tregs, the suppression induced on the effector couple CD4–CD8 increases and therefore the control of tumor development decreases. Tumor cells by themselves secrete TGF-beta, IL-10, VEGF, PGE2 that induce DCs to secrete more TGF-beta contributing to the conversion of CD4(+) T cells to Treg phenotype, enhancing the cellular immune suppression once more. Skin-homing T cells CC-chemokine receptor 4 (CCR4) binds to CCL22 (macrophage-derived chemokine) expressed by tumor-associated macrophages (TAM) and are recruited to the tumor site. On the whole, a favorable microenvironment is created by the concerted action resulting in Tregs’ enhanced proliferation. This action hinders almost completely the CD4–CD8 cooperation and therefore abolishes the activity of antitumoral cytotoxic cells.

Based on our analysis, the *prototype* of the intratumoral inflammatory infiltrate in a melanoma with good prognosis is composed of numerous T cells CD3(+), few/absent B cells CD20(+), few/absent plasma cells and Langerhans cells present^31^. An overview that summarizes the recent findings in this domain is presented in **Table 1**.

**Table 1:** Tissue inflammatory markers

Type	Comments	References
**Cells**		
B lymphocytes (IgMhigh, IgDlow, CD23−, B220low, CD11b+)	Increased in metastasis	^44^
mainly T lymphocytes and dendritic cells (DC)	Correlated with tumor size, stage, metastasis, and patients’ survival	^55^
high peritumoral CD134 (OX40) + and CD25 +	Prognostic factors for longer survival rate	^56^
High peritumoral density of mature DC-LAMP(+) DCs	Significantly longer survival	^50^
density of CD1a(+)	Prognostic impact	
high peritumoral CD1a(+) or DC-LAMP(+) cell density with high number of CD25(+) or OX40(+)	Predictors of good prognosis	
**Molecules**		
HLA-DR, chemokines, NFkappaB p50, MHC II	Markers of invasive primary melanoma, poor prognosis unfavorable clinical outcome	^57^
TRAIL	Marker for stage and for the aggressive/proliferative phenotype	^58^
Low expression of TGF-beta, IL-10, ICAM-1	Prognostic markers for patients	^53^
**Sentinel lymph node**		
Decreased OX40 expression on CD4+ T	Marker for advanced tumor features	^41^

## 4. Inflammatory markers in blood circulation - what do they reflect?

### 4.1. Circulating immune cells

As prognosticators of melanoma evolution, circulatory inflammatory cells are in the spot light of new biomarkers discovery. In this respect, circulating lymphocytes, T, B, NK cells, DCs with immune suppressive phenotype, were some of the recent studied immune cells. **

Our hands-on experience shows that, when drawing the circulatory immune pattern for a melanoma patient,first of all, testing the absolute count of lymphocytes will provide the correct data for detecting the actual circulating subpopulations. Total T CD3(+) lymphocytes is a parameter that will change during the follow-up just in advanced stages and will not give an early prognosticator, while CD4/CD8 ratio will indicate the evolution of the disease and will prognosticate the overall survival of the patient, no matter the stage and the applied therapy.Regarding other circulatory immune cells,we found an increase only in stage III of the circulating percentage of T cells with CD4(+)CD69(+) phenotype indicating a lymph node-related anti-tumoral activity^31^; there are statements that pre-treatment percentages of circulating CD3(+)CD4(+)CD69(+) cells can be an independent prognostic factor for overall survival^59^. Peripheral Tregs increase with stage, but in our investigated group of patients wecould not establish a correlation between the degree of metastasis and the percentages of circulatory Tregs as previously published^60^.

Metastatic melanoma was reported as associated with suppression of Th1 maturation and a Th2 – driven chronic inflammatory state, hence an increased Th2/Th1 ratio. The dominance of Th2 seems to be mediated by tumor-derived VEGF, key player in tumor progression and metastasis. High levels of Th2 cytokines (IL-4, IL-10, IL-13) and chemokines (CCL5 - RANTES, CXCL10) were quantified in metastatic melanoma patient’s plasma, but not in patients with completely resected melanoma (which display a Th1 dominance)^61-63^.

In advanced melanoma patients, immune therapy based on pharmacologically blocking CTLA-4 on Tregs, can be monitored by an increase of circulating CD4(+) and CD8(+) T cell lymphocytes^64,65^.

We have found that advanced stages melanoma show statistically higher circulating CD19(+) B lymphocytes with no increase in plasma level of total Igs and/or Igs subclasses. There is a negative correlation between the level of circulating B lymphocytes and NK cells in melanoma patients^31^.

NK cells have distinct sub-types exerting their immunoregulatory role through target cell lysis (phenotype CD11b^+^ CD27^-^) and/or cytokine production (phenotype CD11b^+/-^ CD27^+^). The ability of NK cells to distinguish between healthy cells and transformed cells (who express insufficient level of MHC class I molecules) and to mediate immune response without prior sensitization is regulated by an integration of signals derived from a complex repertoire of activating and inhibiting receptors as well as various other adhesion and/or costimulatory molecules^66-68^. Although in our studies we did not found circulatory NK levels significantly modified, there are reports showing that melanoma metastatic evolution is associated with an increased frequency of peripheral NK cells expressing receptors for CXCL8, as well as associated with CXCL8 released by tumor tissues^69,70^.

In regional lymph nodes, metastatic melanoma cells activate undifferentiated NK cells and induce generation of CD56^bright^CD16(^+^) and especially CD56^dim^CD16(^+^) NK cell subsets, with highly efficient cytolytic activity compared with blood-derived NK cells^71^. Expression of CD57 increases in terminally differentiated cells with highly cytolytic activity.

CD56^dim^CD57(^+^) activated cells exerts their functions despite the presence of Treg cells. During the progression of melanoma, the CD56^dim^CD57(^+^) / CD56^bright ^CD57(^+^) cells ratio increase (by selective recruitment, expansion or combination of the two processes) and could be used as a prognostic marker in metastatic melanoma^70^.

NK cell subsets seem to play an important role in organ specific susceptibility to melanoma metastasis. Immature CD27^+^CD11b^-^ NK cells seem to protect liver from melanoma metastasis (results reported in a murine model) through a perforin dependent cytotoxic mechanism, while at pulmonary level, more mature subsets CD27^-^CD11b^-^ and CD27^-^CD11b^-^ are responsible for reducing tumor load^72^.

In a recent study, while analyzing various inflammatory factors, it was reported that levels of serum IL-1β, IFN-gamma and CXCL10 were significantly increased in advanced melanoma patients. These circulatory molecules were found correlated with the increased frequency of MDSCs and Tregs. Progression of the disease was associated with an increased serum concentration of IL-1beta and CXCL10 in comparison to the stable disease.

Circulating monocytic (Mo)-MDSCs enhancement was reported as correlated with a decreased progression free survival. This recent study highlights a complex association of circulating inflammatory mediators, Mo-MDSCs and the clinical outcome, hence prognosticators of high risk groups^6^.

The frequency of circulating CD14(+) CD11b(+) HLA-DR(-) /low MDSC correlates with disease progression in patients with different types of cancer, including melanoma. High levels of MDSC were also associated with the absence of T lymphocytes specific for melanoma derived antigens (especially NY-ESO-1 or Melan-A) identified in the peripheral blood of long-term survivors^73^.

We can draw some circulatory immune cells outlines, and one conclusion is that there is no perfect match between circulating immune cells and tumor associated ones, an already acknowledged discrepancy^74^. Another important issue is that circulating immune cell’s phenotype particularities is delicately linked to the stage melanoma is diagnosed in, and that, only the mere broad immune cell population cannot clearly depict the disease evolution. Hence circulatory immune sub-populations, activated and/or suppressor phenotype would give the physician a more focused immune status evaluation of the patient.

### 4.2. Circulatory inflammatory markers

Lactate dehydrogenase (LDH) is the first serum biomarker included in 2001 in American Joint Committee on Cancer (AJCC), biomarker to be used in staging and prognosis evaluation for melanoma patients^75^. The current AJCC guideline still recommends LDH as the only independent biomarker with prognostic value for overall survival for stage IV melanoma patients^76^.

On international level, serum S100B is also in the validation process, and, is a commonly used marker to pinpoint the prognosis of melanoma. A recent study on a large cohort of unresectable stage IV melanoma patients found S100B to be a better independent marker than LDH in terms of prediction of the long-term survival. This could be due to the non-specificity of the abundantly expressed LDH marker, released in the circulatory system in a wide variety of inflammatory disorders associated with cell lysis *versus* the more specific S100B (a molecule secreted by cells originated from the neural crest, including melanocytes or melanoma cells)^77^.

S100B was also linked to inflammation since it interacts with the activated leukocyte cell adhesion molecule (ALCAM) and mediates NF-kB signaling, in a time and dose-dependent manner^78^. Another study brought evidence that circulatory S100B could pinpoint with a better precision than LDH, the poor prognosis in stage IIIB-C melanoma patients^79^.

Our previously published results have shown that in adult melanoma patients, there is a strong correlation between S100B and melanoma inhibitory activity (MIA) and that this association matches an unfavorable clinical evolution. Serum levels of S100B protein were significantly increased in stage IV, in contrast to MIA, where significant increment occurred as early as stage II. We believe that both S100B and MIA are valuable biomarkers for prognosis and therapy monitoring^80^.

A suppression of MIA was obtained *in vitro* when culturing chondrocytes with pro-inflammatory cytokines (TNF-alpha and IL1beta). This fact was clinically sustained when patients with rheumatoid arthritis treated with inhibitors of TNF-alpha and IL1beta showed increased serum levels of MIA^81^. MIA is a protein secreted by chondrocytes and as well as by melanoma cells. Probably the increased level of MIA in melanoma patients with poor prognosis is a potential indicator of pro-inflammatory status switch to a more anti-inflammatory and immunosuppressive status of the disease, but further studies are needed to investigate the actual connection of MIA with inflammation^82^.

Acute phase reactant proteins (APRPs) are usually produced by cytokine-stimulated hepatocytes (e.g. IL-6), these molecules traveling through the bloodstream from the initial site of inflammation. A first response will be obtained in just a few hours and proteins with short half-time (C reactive protein, serum amyloid A) will be secreted by liver. In one-two days, proteins with longer half-time are being synthesized, and are detectable in the serum/plasma for as long as 2 weeks. This pro-inflammatory status is self-limited, but in a wide variety of diseases, including melanoma, prolonged inflammation leads to the persistence of APRPs.

There was a long debate whether to use APRPs as malignancy hallmarks or not, primarily because of their non-specificity. However, traditionally immunoassays and techniques were replaced by modern proteomic analyses, and despite all the challenges, unique combinations of APRPs were recently described for several cancers^83^. Therefore, MALDI-TOF mass spectrometric analysis identifies serum amyloid A as a valuable prognostic marker for all stages of melanoma, with increased specificity and sensitivity for early stages when combined with C reactive protein. Besides the proven superiority over other markers (S100B, LDH), the combination of these two acute phase proteins may have great clinical importance considering the availability and the cost effectiveness of their testing^84^. In addition, ceruloplasmin was found significantly increased in melanoma patients compared with healthy controls^85^.

The initiation of a chronic inflammatory phase can be explained by the immune tolerance developed against the harmful non-self. The tumor escapes the immune system’s action because the pro-inflammatory status is diminished and switched to an immunosuppressive condition. This immune suppression is triggered by a wide variety of mediators, including cytokines, their cell surface receptors, growth factors, matrix metalloproteinase, their inhibitors (TIMP) and acute phase proteins. Investigation of such molecules in the peripheral blood could provide clinicians new valuable prognostic markers for the follow-up of melanoma patients.

High levels of circulating biomarkers associated with poor prognosis in melanoma patients (TNFR2, TGF-alpha, TIMP1, CRP) were recently identified by multiplex technology and described as being part of a valuable formula for overall survival prediction (OS)^86^.

Another study performed on melanoma patients in stage II and III reported the combination of serum TNF-alpha, soluble IL-2 receptor and beta-2 microglobulin to be strong predictive markers of relapse in melanoma before treatment. Among these, only TNF-alpha was able to predict toxicity after treatment with IFN-alpha.

In terms of relapse-free survival, increased serum levels of TNF-alpha seem to have a protective role before and - despite high toxicity - after treatment^87^. The prognostic value of TNF-alpha is still a subject of debate, since past studies have shown no relevant correlation between plasma levels of TNF-alpha and clinical outcome of patients with primary melanoma and negative sentinel lymph node^88^.

Soluble TNF-alpha is a pro-inflammatory cytokine released by cells involved in both innate immune response (macrophages/monocytes) and adaptive immune response (B/T lymphocytes) after proteolytic cleavage of the membrane - bound TNF. The ligand receptors for TNF-alpha, TNF receptor 1 (TNFR1) and TNF receptor 2 (TNFR2), can be membrane–attached or soluble^89^. Inconsistent data regarding the activity of TNF-alpha on tumor cell were presented so far in literature^90^. Recently, several mechanisms were described for TNF-alpha/TNFR2 signaling and new insights were gained regarding context dependent pro-inflammatory/antitumor *versus* anti-inflammatory/protumoral effects of TNF-alpha. It seems that membrane bound TNF-alpha, rather than soluble TNF-alpha, has the ability to activate MDSCs, known to be involved in tumor neo-vascularization. The process is mediated by TNFR2. Stimulated MDSC release an orchestrated cascade of mediators (ARG1, iNOS, NO, ROS, IL-10, TGF-beta) that finally leads to a suppressed immune response^91^. Later studies have shown that membrane-bound TNFR2 is involved, independently of the soluble form^92^. In addition, TNFR2 are also expressed on a subset of Tregs potentiating the anti-inflammatory character and tumor tolerance^93^. Summarizing, it is clear that, TNF-alpha/TNFR2 complex signaling could provide valuable information about the inflammatory/immune status and a better understanding of differences/similarities between membrane-bound and soluble molecules (in terms of expression, functionality, affinity and interactions), insights that could provide novel prognostic markers in this disease.

Our own experience has shown, whether in human samples or mouse melanoma models, that circulatory cytokines have different patterns matching the cutaneous melanoma stages. Thus, in melanoma patients, IL-6 increases with stage, as does TNF-alpha and IL-8; IL-6 being positively correlated with other serum markers tested in our patients, like S100 and MIA^31,94^. IL-6 can pin-point the overall survival of the patient and the circulating levels of IL-1beta can indicate the metastatic processes evolution. Some other cytokines, likeTNF-alpha, IL-8, IL-10 increased only in advanced stage not proving, at least in our group, any discrimination power for early stages.

Regarding our hand-on experience, out of all the tested cytokines, IL-6 level correlated with the patient’s survival, while IL-8, IL-10 and IL-12 did not correlate with overall survival, or relapse-free survival^31^. While we did not find correlation for IL-8 with the overall survival, other authors reported IL-8 as a monitoring biomarker. As a cytokine that is involved in shaping protumoral vascularization and inflammation/immunity, serum IL-8 was found correlated with tumor burden, stage, survival and objective responses to therapy, including those to BRAF inhibitors and immunomodulatory monoclonal antibodies. IL-8 was reported as a potentially useful biomarker to monitor changes in tumor burden following anticancer therapy^95^.

Another recent study brought evidence that circulatory TGF-beta1 can be a good marker for diagnostic and prognostic in melanoma patients. Although higher levels of TGF-beta1 were found in melanoma patients *versus* healthy controls, the responders to therapy and the patients with good clinical outcome in terms of overall survival had elevated levels of TGF-beta1 compared to non-responders and compared to patients with poor prognosis^96^. This study conveys new up-dated insights after past controversies on TGF-beta1 role in skin inflammation^97^.

The wide variety of circulatory mediators and cells involved in the complex switch between acute and chronic inflammation could provide new early indicators of the tumor immunosuppressive status induced by prolonged inflammation and could bring new information in early diagnostic of cutaneous melanoma, aid in prognosis evaluation and therapy monitoring (summarized in **Table 2**).

**Table 2:** Inflammatory markers in blood circulation

Type	Comments	References
**Cells**		
CD3+CD4+CD69+ T cells	Independent prognostic factor for overall survival	^59^
CD19+ B lymphocytes	High levels found in patients with advanced stage	^31^
Peripheral NK cells	Increased in metastatic melanoma	^69,70^
Circulating monocytic (Mo)-MDSCs	Correlated with disease progression	^6^
**Molecules**		
LDH	Staging and prognosis marker	^76^
S100B, MIA	Common used markers for prognosis and monitoring therapy	^80^
Serum amyloid A, C reactive protein	Acute phase reactant protein	^84^
Ceruloplasmin	Acute phase reactant protein	^85^
TNFR2, TGF-alpha, TIMP1, CRP	Mediators of chronic inflammatory, indicators of immunosuppressive status	^86^
TNF-alpha, soluble interleukin-2 receptor and beta-2 microglobulin	Predictive markers of relapse	^87^
IL-6, IL-8, TNF-alpha	Cytokines, staging markers	^31^
IL1-beta	Marker of metastasis	^31^
TGF-beta1	Growth factor involved in skin inflammation, marker for diagnosis and prognosis	^97^
Cytokines IL-4, IL-10, Il-13 and chemokines CCL5(RANTES), CXCL10	High levels of Th2 cytokines in patient plasma with metastatic melanoma	^61-63^

## 5. Conclusive remarks

Acute inflammation has a good purpose, being beneficial through its final goal of healing the damaged tissue. It is the chronic inflammation that triggers the molecular pathways to an immunosuppressive status, which clinically translates to a poor prognosis in melanoma patients. Detecting the fine switch from acute to chronic inflammation as early as possible could provide powerful predictive markers for clinical outcome in melanoma patients and early markers for diagnosis.

A panel of tissue/circulatory immune markers can complete the immune status of the patient, can add value to the overall prognostic of the patient and thus direct/redirect the therapy choice. The future lies within establishing low-cost, affordable/available, easily reproducible assays that will complete the pre-clinical parameters of the patient.

## Bullet Points

- **The molecular pathways of melanoma-related inflammation are now being unveiled, insights that can lead to identification of new target molecules, hence improved diagnosis and treatment.**
- **Discovering that chronic inflammation contains the “seeds” for possible tumorigenesis events opens the therapeutical possibilities that can favor adaptive immunity instead of tumor development.**

## Open Questions

- **What is the clinical relevance of the connections between sex steroid hormones and inflammation?**
- **Is there a still unknown intercellular communication between myeloid – derived suppressor cells and tumor associated macrophages?**
